# Extended High‐Risk HPV Genotyping With BD Onclarity Enhances Anal Cancer Screening Among High‐Risk Populations: A Cross‐Sectional Study

**DOI:** 10.1002/dc.70160

**Published:** 2026-06-17

**Authors:** Daisy Maharjan, Joshua Waters, Melissa Randolph, Benjamin Shuler, Julie Borum, Sheila Segura

**Affiliations:** ^1^ Department of Pathology and Laboratory Medicine Indiana University School of Medicine Indianapolis Indiana USA; ^2^ Indiana University Health General Surgery–Downtown Indianapolis Indianapolis Indiana USA; ^3^ Indiana University Health LifeCare Indianapolis Indiana USA

**Keywords:** anal cancer, cytology, high‐risk HPV, high‐risk population

## Abstract

**Background:**

Anal cancer rates are rising among women, immunocompromised individuals, and Men who have sex with Men (MSM), independent of HIV status. While high‐resolution anoscopy remains the diagnostic standard, limited access has increased interest in alternative screening. HPV testing now complements or replaces cytology in cervical cancer prevention, but evidence for anal screening, especially using extended genotyping platforms like BD Onclarity, remains limited.

**Methods:**

This single center cross‐sectional study enrolled high‐risk individuals undergoing anal cancer screening from May 2024 to May 2025. Anal cytology and high‐risk HPV (HR‐HPV) DNA testing were performed using the BD Onclarity assay on the BD COR system. Demographics, cytology results, HRHPV genotypes, and available histopathology were analyzed.

**Results:**

Among 117 high‐risk participants (median age 42; 106 males, 8 females, 3 transgender women), 74.3% were ‐HIV positive, predominantly MSM‐. Abnormal cytology occurred in 41% of cases including ASC‐US (*n* = 29; 60.4%), LSIL (*n* = 16; 33.3%), ASC‐H (*n* = 2; 4.1%), and HSIL (*n* = 1; 2.1%). Valid BD Onclarity results were available for 104 individuals, with 61.5% testing HR‐HPV positive. Non‐16/18 HR‐HPV types were most common overall, while HPV16 predominated in abnormal cytology cases. Twelve individuals underwent biopsy, revealing no dysplasia (*n* = 2), AIN1 (*n* = 5), AIN2 (*n* = 1), and AIN3 (*n* = 4).

**Conclusions:**

Extended HR‐HPV genotyping using the BD Onclarity assay demonstrated a high prevalence of HR‐HPV in this high‐risk population, with multiple genotype infections commonly observed. While non‐HPV16/18 genotypes predominated overall, HPV16 was more frequently associated with abnormal cytologic and high‐grade histologic findings. Given the limited number of biopsy‐confirmed high‐grade lesions, these associations should be interpreted as descriptive and hypothesis‐generating. Cytology remains central to anal cancer screening, with extended HPV genotyping serving as a complementary adjunct rather than a standalone stratification tool. Larger longitudinal studies with systematic histopathologic follow‐up are needed to clarify the clinical significance of extended HPV genotyping in this population.

## 1 Introduction

Anal cancer accounts for approximately 0.5% of all newly diagnosed cancers in the United States, with an estimated 10,930 new cases and 2030 deaths projected in 2025 [1]. While the incidence of anal cancer is low in the general population, its incidence and mortality have risen significantly among individuals living with HIV and men who have sex with men (MSM). HIV‐positive MSM are at the highest risk, with incidence rates reported between 80 and 131 per 100,000 person‐years [2, 3, 4]. This elevated risk is largely attributed to immunosuppression and persistent infection with high‐risk HPV (HR‐HPV). Additional risk factors include a history of anal warts, previous HPV‐associated malignancies (cervical, vaginal, or vulvar cancer), smoking, and certain sexual behaviors such as having multiple sexual partners and receptive anal intercourse [5].

Similar to cervical cancer, the development of anal cancer is strongly associated with persistent HR‐HPV infection. Both diseases originate in transformation zones vulnerable to HPV‐mediated dysplasia and demonstrate parallel progression from precursor lesions, cervical intraepithelial neoplasia (CIN2‐3) in the cervix and anal intraepithelial neoplasia (AIN2‐3) in the anus [6]. Established cervical cancer screening strategies, including cytology, HR‐HPV testing, and co‐testing, have successfully reduced cervical cancer incidence and mortality [7, 8]. These prevention successes have motivated the application of similar screening principles to the anal canal, particularly for individuals at highest risk.

A major advancement in this field was the ANCHOR (Anal Cancer/HSIL Outcomes Research) trial, a pivotal randomized controlled study demonstrating that routine detection and treatment of anal high‐grade squamous intraepithelial lesions (HSIL) significantly reduces progression to anal cancer in people living with HIV [9]. These findings support the implementation of structured anal cancer screening programs modeled after cervical cancer prevention strategies.

In response to this evidence, the International Anal Neoplasia Society (IANS) published comprehensive 2024 guidelines outlining screening approaches for high‐risk groups. Recommended strategies include: (1) anal cytology with HR‐HPV triage, (2) primary HR‐HPV testing with cytology triage, and (3) cytology and HR‐HPV co‐testing [10]. However, evidence supporting a preferred strategy remains limited. Although HR‐HPV testing of anal specimens is not FDA‐approved, it has been shown to be a highly sensitive tool for detecting anal precancer and cancer [11].

Despite growing interest, studies evaluating HR‐HPV testing for anal cancer screening remain relatively sparse and often predate the IANS guidelines. Even fewer studies have examined extended HPV genotyping, beyond HPV16 and HPV18, in anal cytology specimens. Our institution implemented extended HR‐HPV genotyping in December 2022, initially using the Cobas assay before transitioning in May 2024 to the BD Onclarity HPV assay, which allows for detection of additional high‐risk genotypes.

In this study, we aim to evaluate the performance of the BD Onclarity HPV assay in detecting HR‐HPV in anal specimens collected from high‐risk individuals. We also assess the correlation between HR‐HPV detection and anal cytology results to inform potential strategies and contribute evidence to an emerging field with evolving best practices.

## 2 Materials and Methods

The study was approved by the Institutional Review Board of the Indiana University School of Medicine and conducted as a single‐center, cross‐sectional descriptive analysis. The study population consisted of individuals at high risk for anal cancer who underwent routine anal cancer screening between May 2024 and May 2025. Screening procedures included anal cytology and HPV DNA testing using the BD Onclarity HPV assay. Specimens for both tests were collected during standard clinic visits. Demographic and clinical variables were also collected, including age, gender, ethnicity, HIV status, relevant risk factors, HPV vaccination status, prior anal screening results, and follow‐up anoscopy and biopsy findings when available.

Anal swabs were collected using Dacron swabs and placed into BD SurePath vials containing liquid‐based fixative. Samples were processed with the BD SurePath Totalys Multiprocessor. Cytology slides were prepared according to standard laboratory protocols and interpreted by board‐certified cytopathologists using the 2014 Bethesda System for Reporting Cervical Cytology. Cytologic diagnoses included: nondiagnostic, negative for intraepithelial lesion or malignancy (NILM), atypical squamous cells of undetermined significance (ASC‐US), low‐grade squamous intraepithelial lesion (LSIL), atypical squamous cells, cannot exclude HSIL (ASC‐H), high‐grade squamous intraepithelial lesion (HSIL), or carcinoma [12].

Although the BD Onclarity HPV assay is FDA‐approved for cervical specimens only, it was analytically validated at our institution for detection of high‐risk HPV DNA in anal specimens prior to clinical use. This assay employs Real‐Time Polymerase Chain Reaction (PCR) technology to target E6 and E7 viral oncogenes for the detection of 14 HR‐HPV genotypes, reporting six individually (HPV 16, 18, 31, 45, 51, and 52) and eight in three pooled groups (HPV 33/58, 56/59/66, 35/39/68).

Analyses were descriptive; inferential statistics were not performed due to small sample size for histologic outcomes.

## 3 Results

A total of 117 participants were enrolled, ranging in age from 24 to 68 years (median: 42 years). The cohort was predominantly male (*n* = 106), with eight females and three transgender women. Racial and ethnic distribution included: 42% White non‐Hispanic, 41% African American, 3.4% Hispanic, 1.7% Asian, and 12% unspecified (see Table 1). Overall, 87 participants (74.3%) were living with HIV.

**TABLE 1:** Demographic and risk factors of the study population.

Gender	N (%)
Male (M)	106 (90.5%)
Female (F)	8 (6.8%)
Transgender women (TW, MTF)	3 (2.5%)
Race/ethnicity	
Black or African American	48 (41%)
White (non‐Hispanic)	49 (42%)
Hispanic	4 (3.4%)
Asian	2 (1.7%)
Not specified	14 (12%)
Risk factors	
MSM with HIV	80 (68.3%)
MSM without HIV	23 (19.6%)
MSM with HIV and IVDU	4 (3.4%)
Heterosexual females with HIV	2 (1.7%)
Heterosexual females with HPV‐related gynecologic lesions (HIV‐negative)	4 (3.4%)
Heterosexual females without HIV	2 (1.7%)
Not specified	2 (1.7%)

Abbreviations: IVDU, intravenous drug use; MSM, Men who have sex with men; TW, Transgender women.

The largest subgroup consisted of 80 men who have sex with men (MSM) living with HIV. An additional 4 MSM were living with HIV and had a history of intravenous drug use (IVDU), and 23 MSM were HIV‐negative. For analytical consistency, 3 transgender women (TW) were grouped within the MSM category. Eight participants identified as heterosexual females; 2 were HIV‐positive and 6 were HIV‐negative. Among the HIV‐negative females, 2 had persistent high‐risk cervical HPV infections and 2 had a history of high‐grade vulvar intraepithelial neoplasia (VIN3). Two HIV‐negative females presented with nonspecific risk factors (anal hemorrhoids and bleeding). In two cases, risk factors were unspecified (see Table 1).

Regarding HPV vaccination status, 58 participants had not received vaccination, 39 had completed the full vaccination series, and 20 were partially vaccinated.

### 3.1 Anal Cytology Findings

Anal cytology was performed in all 117 cases. Abnormal cytology was identified in 48 cases (41%) cases, comprising 29 ASC‐US (60.4%), 16 LSIL (33.3%), 2 ASC‐H (4.1%), and 1 HSIL (2.1%). Cytology was negative (NILM) in 66 cases (56.4%), and 3 cases (2.5%) were nondiagnostic (see Table 2).

**TABLE 2:** Correlation of HR‐HPV test results with cytology findings.

HR‐HPV result	Cytology findings						Total
	NILM	ASCUS	ASC‐H	LSIL	HSIL	Non‐diagnostic	
HPV16	2	1		1			4
HPV18		1		1			2
HPV16,18				1			1
HPV16,18, OTHER		1		1			2
HPV16, OTHER	3	4		4	1		12
HPV35/39/68	6	4					10
HPV56/59/66	3	2					5
HPV 51	4			1			5
HPV33/58	2			1			3
HPV52		1				1	2
HPV45						1	1
HPV35/39/68 + Other HR‐HPV	3	5	1	2			11
Multiple other HR‐HPV combinations	1	2	1	2			6
Negative	31	7		2			40
Not done/invalid	11	1				1	13
Total	66	29	2	16	1	3	117

Abbreviations: AIN, Anal intraepithelial neoplasia; ASC‐H, Atypical squamous cells, cannot exclude HSIL; ASCUS, Atypical squamous cells of undetermined significance; HSIL, High‐grade squamous intraepithelial lesion; LSIL, low‐grade squamous intraepithelial lesion; NILM, Negative for intraepithelial lesion or malignancy.

Among the 48 cases with abnormal cytology, 21 represented newly diagnosed abnormalities with no prior screening history. The remaining 27 had a documented history of prior anal cytology. Sixteen of these had prior abnormal cytology, with four showing progression: one from LSIL to HSIL, two from ASC‐US to LSIL, and one from ASC‐US to ASC‐H. Eleven cases had prior normal cytology; among these, 5 demonstrated newly detected HR‐HPV infections compared to previous Cobas HPV testing.

Among cases with stable cytology, 11 participants had prior biopsies: 2 were normal and 9 showed AIN1. These patients continued routine annual anal cytology screenings.

### 3.2 HR‐HPV Test Findings

BD Onclarity HPV testing was performed in 109 cases, of which 5 yielded invalid results. Of 104 valid results, HR‐HPV was detected in 64 cases (61.5%). Single‐genotype infections were identified in 32 cases (50%), while the remaining 32 cases (50%) showed multiple genotype infections. The most frequently detected category included HR‐HPV genotypes other than HPV16 and HPV18, grouped as “Other HR‐HPV”, comprising 43 cases in total.

Within the Other HR‐HPV category, detected genotypes included HPV31, HPV45, HPV51, HPV52, and pooled groups HPV33/58, HPV56/59/66, and HPV35/39/68. Among these, 26 were single‐genotype infections, and 17 were multiple‐genotype infections. HPV35/39/68 was the most commonly identified pooled genotype group, with 9 single infections and 12 combined infections. Cytologic correlation for HPV35/39/68 positive cases included 8 NILM, 10 ASC‐US, 2 LSIL, and 1 ASC‐H (see Table 2).

Following HPV35/39/68, HPV16 was the next most common genotype, identified as a single infection in 4 cases, in combination with HPV18 in 1 case, and in combination with other HR‐HPV genotypes in 12 cases. Cytologic correlation for HPV16 positive cases demonstrated 5 NILM, 6 ASCU‐S, 7 LSIL, and 1 HSIL (see Table 2).

### 3.3 Anoscopy and Biopsy Findings

Of the 48 cases with abnormal cytology, 21 newly diagnosed abnormalities, 11 cases with prior normal cytology, and 4 cases showing cytologic progression were referred to colorectal surgery for high‐resolution anoscopy (HRA). Nineteen patients did not attend follow‐up. Among those evaluated, 2 demonstrated mild acetowhite changes that were treated with ablation; three had normal anoscopic findings, and 12 underwent biopsy.

Histopathologic evaluation of the 12 biopsied cases revealed 4 cases of anal intraepithelial neoplasia grade 3 (AIN3), one case of AIN2, five cases of AIN1, and two cases with no evidence of dysplasia. Among the four AIN3 cases, 3 were positive for HPV16 in combination with other HR‐HPV genotypes, including HPV18, HPV31, HPV56/59/66, HPV52, and HPV35/39/68. The remaining AIN3 case was positive for HPV35/39/68 alone. The single case of AIN2 demonstrated a mixed infection with HPV51, HPV52, and HPV56/59/66 (see Table 3).

**TABLE 3:** Clinical profile, cytologic and HPV findings in 14 cases with available biopsy results.

Case number	Age	Sex	Risk factor	HIV status positive	Cytology	HR‐HPV	Anal biopsy
1	69	M	MSM	Y	ASCUS	HPV31, HPV35/39/68	AIN1
2	42	M	MSM	Y	ASCUS	HPV16, HPV31, HPV56/59/66,52	AIN1
3	61	M	MSM	Y	LSIL	HPV16, HPV33/58, HPV 35/39/68	AIN1
4	34	M	MSM	Y	ASCUS	HPV 16	AIN1
5	29	M	MSM	Y	ASCUS	HPV31, HPV52	AIN1
6	50	F	VIN3	N	ASC‐H	HPV51, HPV52, HPV56/59/66	AIN2
7	33	F	VIN3	N	LSIL	HPV16, HPV18	AIN3
8	46	M	MSM	N	ASCUS	HPV16, HPV56/59/66	AIN3
9	64	F	Nonspecific	N	ASCUS	HPV35/39/68	AIN3
10	37	M	MSM	Y	ASCUS	HPV16, HPV31, HPV56/59/66, HPV52, HPV35/39/68	AIN3
11	52	M	MSM	Y	ASCUS	HPV52	Negative for dysplasia
12	29	M	MSM	Y	LSIL	HPV31, HPV33/58	Negative for dysplasia
13	63	M	MSM	Y	NILM	HPV16	Negative for dysplasia
14	43	M	MSM	Y	NILM	HPV16	Negative for dysplasia

Abbreviations: AIN, Anal intraepithelial neoplasia; ASC‐H, Atypical squamous cells, cannot exclude HSIL; ASCUS, Atypical squamous cells of undetermined significance; F, Female; LSIL, low‐grade squamous intraepithelial lesion; M, Male; MSM, Men who have sex with men; N, No; NILM, Negative for intraepithelial lesion or malignancy; Y, Yes.

Among the 5 AIN1 lesions, HPV16 was detected in three cases, 1 as a single‐genotype, and 2 in combination with other HR‐HPV types (HPV31, HPV56/59/66, HPV52, HPV33/58, and HPV35/39/68). One additional AIN1 case was positive for HPV31 and HPV35/39/68, and another showed co‐infection with HPV31 and HPV52 (see Table 3).

Among HP16 positive cases with NILM cytology, biopsy was performed in two individuals and showed no evidence of dysplasia; the remaining HPV16 positive/NILM cases did not undergo histologic evaluation.

## 4 Discussion

Persistent infection with Human papillomavirus (HPV) is central to the pathogenesis of anal cancer. Until early 2024, anal cancer screening lacked standardized, widely accepted clinical guidelines. This changed with the International Anal Neoplasia Society (IANS) 2024 guidelines, which stratified high‐risk individuals into two categories based on relative anal cancer incidence. Risk Category A includes groups with ≥ 10‐fold increased risk, such as MSM and transgender women (TW) with HIV, women with HIV, men who have sex with women (MSW) with HIV, MSM and TW without HIV, individuals with prior vulvar HSIL or cancer, and solid organ transplant recipients. Risk Category B includes groups with up to a 10‐fold increased risk, including individuals with cervical or vaginal HSIL or cancer, persistent cervical HPV16 infection, perianal warts (male or female), and other immunosuppressive conditions. In our study, MSM living with HIV constituted the majority, consistent with known epidemiology and explaining the high HR‐HPV prevalence observed.

Much of the existing literature on anal precancer and cancer focuses on MSM living with HIV, who carry the highest risk for anal squamous cell carcinoma [4, 13]. Prior studies consistently demonstrate a high prevalence of HR‐HPV in this population [14, 15, 16, 17, 18, 19, 20].

According to IANS guidelines, anal cytology alone is an acceptable screening method, with high‐resolution anoscopy (HRA) recommended for abnormal results. When HRA capacity is limited, only high‐grade cytology warrants immediate referral; other abnormalities may be re‐evaluated in 12 months. HR‐HPV testing, with or without genotyping, can be used as a triage tool to limit the number of immediate HRA referrals. Primary HR‐HPV testing is also acceptable, with positive results prompting HRA or repeat screening in 12–24 months if negative [10].

However, in high‐risk populations such as MSM with HIV, the extremely high prevalence of anal HPV limits the specificity of HPV testing, potentially increasing HRA referrals. While this is challenging in resource‐limited centers, HR‐HPV testing can still serve as a valuable triage tool where cytology is unavailable or where universal HRA is otherwise performed [10].

In our study, although HR‐HPV prevalence was high, cytology was normal in more than half of participants. Only those with abnormal cytology were referred for HRA and biopsy. Among HR‐HPV positive cases, the most common genotypes overall were non‐HPV16/18 (Other HR‐HPV), particularly the pooled HPV35/39/68 group, followed by HPV16. This distribution aligns with the predominance of normal and low‐grade cytology results. Prior studies have consistently shown HPV16 to be strongly associated with high‐grade anal lesions and anal cancer, while other HR‐HPV types are more frequently found in low‐grade lesions and decline in prevalence in invasive cancer [18, 21, 22].

When considering only cases with abnormal cytology, HPV16 became the most prevalent genotype, a finding consistent with earlier work [23, 24, 25]. Literature on anal HPV genotyping is heterogenous, owing to differing study populations and assay platforms. Importantly, the BD Onclarity assay does not distinguish HPV35, HPV39, and HPV68 individually, which limits direct comparison with studies using assays that identify these types separately. Other studies using assays such as Anyplex HPV14 (Seegene) or Atila Biosystems multiplex testing have identified varying genotype distributions, including HPV52, HPV68, and HPV53, reflecting both assay differences and population characteristics [23, 24].

Only a subset of participants returned for follow up HR and biopsy, consistent with known challenges in maintaining engagement in anal cancer screening among people with HIV. Barriers include lack of awareness, low perceived benefit, stigma, socioeconomic challenges, and logistical limitations [26].

Although HPV16 was the most frequently detected genotype in cases with high grade histology/AIN3, these findings represent observed patterns within this cohort and should be viewed as hypothesis‐generating rather than definitive evidence of genotype‐specific risk. Other HR‐HPV types were primarily associated with normal biopsy findings or AIN1, although one AIN3 lesion was linked to HPV35/39/68. Importantly, cases with normal cytology but HPV16 positivity were not referred for HRA; this may have resulted in missed high‐grade lesions. Other studies have shown that a significant proportion of HPV16 positive individuals with normal cytology may harbor HSIL on HRA, highlighting an area for potential refinement of screening strategies [23].

The oncogenic contributions of non‐HPV16 HR‐HPV genotypes in anal cancer are less well understood than those of HPV16. According to the study by K Wong et al., non‐HPV 16/other HR‐HPV were encountered more frequently in squamous cell carcinoma from immunodeficient individuals compared to immunocompetent individuals, hinting on oncogenic potential of these genotypes in MSM with HIV [21]. Recent study in MSM living with HIV have reported associations between anal HSIL and HPV33, HPV35, and HPV56 [27]. Other work has also documented the presence of HPV33, HPV35 and HPV51 in anal squamous cell carcinoma [28, 29]. Although studies have shown that non‐HPV16 genotypes are prevalent in anal precursor lesions and anal cancer, underscoring the need for improved risk stratification, the frequent occurrence of multiple co‐infections complicates establishing a clear causal relationship of individual genotype [16, 30].

## 5 Novelty and Contribution to the Field

This study provides important real‐world data on the implementation of extended HPV genotyping using the BD Onclarity assay for anal cancer screening, an area where evidence remains limited. While extended genotyping is well established in cervical screening, its application in anal specimens is understudied, and few published studies evaluate assay‐specific genotype distributions using BD Onclarity. Our findings demonstrate that: 1. Extended HR‐HPV genotyping is feasible in routine anal screening workflows, 2. Non‐HPV16 HR‐HPV types, particularly the pooled HPV35/39/68 group, are common in high‐risk populations, and 3. HPV16 remains the strongest correlation of abnormal cytology reinforcing its critical role in risk stratification. This contributes to new evidence supporting the integration of extended genotyping into anal screening, particularly in settings adopting the 2024 IANS guidelines.

## 6 Limitations

Our study has several limitations. It is cross‐sectional in design and includes a limited number of high‐resolution anoscopy‐guided biopsies. Biopsy was performed only when clinically indicated based on abnormal cytology and anoscopic findings; cases with normal anoscopy, stable cytologic abnormalities with prior biopsy, or failure to attend follow‐up were not re‐biopsied. Follow‐up was further limited by patient nonattendance and the relatively short study period. As a result, the number of histologic specimens was small, limiting robust genotype–outcome correlation, and histopathologic associations should be interpreted as descriptive rather than inferential. Additionally, because HPV16‐positive cases with normal cytology were not routinely referred for high‐resolution anoscopy, occult high‐grade lesions in this subgroup cannot be excluded.

## 7 Conclusion

This study demonstrates a high prevalence of high‐risk HPV infection in a high‐risk population undergoing annual cancer screening and shows that extended HPV genotyping using the BD Onclarity assay is feasible in routine clinical practice. While the high prevalence of HPV in this population limits the specificity of genotyping alone, HPV testing provides complementary information when interpreted alongside anal cytology findings.

Non‐HPV 16/18 high‐risk genotypes were frequently detected in individuals with normal or low‐grade cytology, whereas HPV 16 was more commonly observed in cases with abnormal cytology and high‐grade histology. However, given the limited number of biopsy‐confirmed high‐grade lesions and the descriptive nature of this study, these genotype associations should be interpreted as hypothesis generating rather than indicative of definitive genotype‐specific risk.

Importantly, our findings reinforce the central role of anal cytology and screening decision making within high‐risk populations. Extended HPV genotyping may enhance cytologic assessment but does not replace it, particularly in settings where access to high‐resolution anoscopy is limited. This study was not designed to establish genotype‐based risk stratification or clinical management thresholds, and no changes to current screening or referral practices are proposed based on these findings.

Larger longitudinal studies with systemic histopathologic follow‐up are needed to better define the clinical significance of extending extended HPV genotyping, particularly for non‐HPV16 genotypes, and to inform and refine screening strategies.

## Author Contributions

**Daisy Maharjan:** writing – review and editing, writing – original draft, data curation, formal analysis. **Joshua Waters:** writing – review and editing, resources, data curation. **Melissa Randolph:** writing – review and editing, methodology, formal analysis, data curation. **Benjamin Shuler:** writing – review and editing, methodology, formal analysis, data curation. **Julie Borum:** resources, data curation. **Sheila Segura:** conceptualization, supervision, methodology, writing – review and editing.

## Funding

The authors have nothing to report.

## Conflicts of Interest

The authors declare no conflicts of interest.

## Data Availability

The data that support the findings of this study are available from the corresponding author upon reasonable request.
